# Quality of life and associated factors among people receiving second-line anti-retroviral therapy in Johannesburg, South Africa

**DOI:** 10.1186/s12879-022-07429-9

**Published:** 2022-05-12

**Authors:** Nomcebo Oratile Mokgethi, Nicola Christofides, Mercilene Machisa, Godspower Akpomiemie, Samantha Lalla-Edward

**Affiliations:** 1 Epidemiology and Biostatistics, WHO, Bloemfontein, Free State South Africa; 2 School of Public Health, Wits University, Johannesburg, South Africa; 3 Ezintsha, Faculty of Health Sciences, University of the Witwatersrand, Johannesburg, South Africa; 4 Gender and Health Research Unit, South African Medical Research Council, Pretoria, South Africa

**Keywords:** Quality of life, Anti-retroviral treatment, HIV drug effects, South Africa, Darunavir

## Abstract

**Background:**

Studies which examine quality of life (QOL) provide important insights that are needed to understand the impacts of HIV/AIDS anti-retroviral treatment (ART), comorbid conditions and other factors on the daily activities of people living with HIV/AIDS (PLH). This study aimed to determine the inter-relationships between clinical factors, behavioural, socio-demographic variables and QOL among PLH.

**Methods:**

The secondary analysis used data collected from 293 people living with HIV/AIDS (PLH) receiving second-line ART in Johannesburg in a clinical trial which evaluated the non-inferiority of ritonavir-boosted darunavir (DRV/r 400/100 mg) compared to ritonavir-boosted lopinavir (LPV/r) over a 48 week-period. Physical functioning, cognitive and mental QOL were measured using the Aids Clinical Trial Group questionnaire. Exploratory factor analyses were used to examine the structure, the relationships between and the construct validity of QOL items. Structural equation models which tested the a priori-hypothesised inter-relationships between QOL and other variables were estimated and goodness of fit of the models to the data was assessed.

**Results:**

Patients on darunavir presented with lower pill burden. Older patients and women were more likely to report lower QOL scores. Pill burden mediated the effects of age, sex and treatment regimen on physical functioning QOL and adverse effects; the effects of age, sex, treatment regimen and adverse effects on cognitive QOL; and the effects of sex on mental QOL.

**Conclusion:**

QOL among PLH is associated with socio-demographic and clinical factors. Therefore, QOL could be enhanced by considering PLH characteristics, clinical factors such as regimen side-effects profile, management of comorbid conditions and mitigating risks such as potential adverse drug-to-drug interactions among patients on ART.

**Supplementary Information:**

The online version contains supplementary material available at 10.1186/s12879-022-07429-9.

## Introduction

Quality of life (QOL) which encompasses an “individual’s perception of their position in life in the context of the culture and value systems in which they live and in relation to their goals, expectations, standards, and concerns” [1], is an important measure in therapeutic research and practice. The concept of QOL is multi-faceted, reflecting different dimensions and experiences in a person’s life, such as their psychological, social and physical function. It focuses on assessing functional changes that may occur overtime in various illnesses [2, 3]. Measuring QOL and determining associated factors has become important for understanding patients’ experiences of living with chronic diseases, and how it affects daily activities [3].

Global advancements in the delivery of anti-retroviral therapy (ART) to people living with HIV/AIDS (PLH) has decreased mortality: PLH can live longer, albeit with their QOL negatively impacted by health-challenges related to the clinical manifestation of HIV, the adverse events of long-term exposure to treatment or emerging co-morbidities related to HIV [4–7]. Therefore, measuring QOL and determining associated factors are necessary for understanding impact of disease and targeting interventions that will mitigate the burden on individuals and the health system altogether [6, 8]. Additionally, findings from the study will assist healthcare practitioners and HIV programs to not only monitor the physical well-being of their patients but other QOL domains may be affected such as mental and social well-being [2].

Several studies have investigated worldwide factors that affect QOL among PLH [5, 9, 10]. Previous research has found concomitant medication administered for presence of co-morbidities impact QOL among PLH [11–13]. Co-morbid conditions increase the risk of adverse effects and complex medication regimens administered in attempts to manage these conditions may lead to additive toxicities and further increase adverse events and morbidity [14]. Moreover the compounded HIV-related challenges increase clinic visits and hospitalizations that lead to higher usage of healthcare resources by PLH [6, 15]. Other factors associated with QOL among PLH are behavioural in nature for example adherence to treatment [16, 17] and severe substance abuse [18, 19]. Lastly, biological sex of the patient, stigma and discrimination have also been reported to impact QOL [20, 21].

The QOL in HIV patients initiated on first-line ART varies according to disease severity, demographics and geography/country and for most patients tends to improve after starting ART [5, 22]. Consistent use of ART facilitates viral suppression which in turn improves QOL of PLH. However, sometimes patients develop resistance to treatment, making the virus unsuppressed and continuously replicating [23, 24]. In a study by Torres and colleagues, using the ACTG SF-21 tool, individuals failing first-line ART showed lower QOL in most domains except role function and social function [25]. The literature on first-line failures in South Africa is scanty, yet show that over 2% (which translates to over 10,000 of HIV patients) switch to second-line therapy annually [26].

While previous studies have investigated the factors associated with QOL among PLH, scholars used traditional regression models which are limited in elucidating the inter-relationships among the different factors. However, the application of novel structural equation modelling in understanding QOL allows the critical investigation of the direct and indirect pathways that affect QOL among PLH. This study was conducted to determine the inter-relationships among clinical factors, including severity of adverse events, pill burden (defined as medication taken in addition to the ART regimen) and ART treatment regimen, behavioural and socio-demographic variables and QOL among PLH receiving second-line ART in Johannesburg. Our a priori hypothesis assumed that adverse events [frequency and severity (clinical factor)] impacted on pill burden (clinical factor) and QOL; greater frequency and severity of adverse events increases pill burden (mediator) but would reduce QOL; treatment regimen (clinical factor) could impact on QOL through direct and indirect paths mediated by adverse events and pill burden and that socio-economic status could directly impact on QOL or this relationship could be mediated by substance use (behavioural).

## Methodology

### Study population: primary study

This secondary analysis used data collected from participants with HIV in a randomized control trial (RCT) conducted by the Wits Reproductive Health and HIV Institute (Wits RHI). The RCT investigated non-inferiority of ritonavir-boosted darunavir (DRV/r 400/100 mg) compared to the current standard second-line therapy ritonavir-boosted lopinavir (LPV/r) among 300 adults, HIV-1 positive patients receiving second-line ART in Johannesburg over 48 weeks (Protocol number: WRHI052). Ethics clearance was obtained from the l Human Research Ethic Committee (Medical) (M181001). A more detailed description on the participant selection criteria and procedures can be found elsewhere [27]. Of the 300 participants, this secondary data analysis included 293. We excluded seven participants due to early withdrawal (before week 48) (n = 4), relocation (n = 1), death (n = 1) and protocol deviation (n = 1).

### Data collection and key measures

#### Variable measurement and recoding

This secondary data analysed was collected through the mentioned primary study whose methods and variable measurement are described in detail elsewhere [27]. The primary study measured explanatory and demographic variables namely age, sex, race, marital status, education, and employment status. Patient demographic and other details were collected at the screening visit and recorded on a screening form. Participants’ second-line therapy was informed by their randomisation to either the lopinavir (5 pills) or the darunavir (3 pills) treatment arm at enrolment visit. Pill burden counts were defined as medication taken additional to ART administered. The medication was taken from a concomitant medication source document that was regularly updated at every study visit. The number of co-medications was counted to give the total number of medications a participant self-administered over 48 weeks. Therefore, the co-medication/pill burden was used as a proxy indicator for co-morbid conditions that patients were facing. Adverse events were recorded at unscheduled and scheduled study visits. The total number of adverse events experienced over the 48 weeks period was categorised based on the Division of AIDS grading table used in the primary study (version 2.0, November 2014) [28] i.e., mild symptoms (causing no or minimal interference with usual social and functional activities), moderate (causing greater than minimal interference with usual social and functional activities), and severe or life-threatening (causing inability to perform basic self-care functions). If a patient presented with more than 1 adverse event, the highest grade was allocated to that patient. The events were then grouped together to form one variable. Data pertaining to alcohol, smoking tobacco, and illicit drug use were also extracted at the screening visit.

The primary study measured QOL using the standard ACTG-SF 21 (601-2) health survey questionnaire and manual administered by a research nurse at enrolment and at 5 follow up visits from Week 4 to Week 48, with Week 48 being the end of study visit (EOS) [29, 30]. In this study, QOL domains at Week 48 were used as the study outcomes. The original questionnaire consisted of eight domains and 17 questions which focused on general health perceptions, physical function, role function, pain, social function, mental health, energy, and cognitive function (Additional file 1: Appendix A). Table 1 shows how measured items in each domain were recoded by summing responses to create sub-domain scores before transforming each score to a scale ranging from 0 to 100 (Table 1). Higher transformed scores on the scales indicated better health.

**Table 1:** Description and transformation of the ACTG QOL domains

Domains	Items	Item example	Scale	Transformation equation
General health perception	1	In general, would you say your health is?	1–5 (poor = 1 to excellent = 5)	= (100/(5 − 1)) * (general health perception raw score − 3)
Pain	1	How much bodily pain have you had in the past 4 weeks?	1–5 (none = 1 to severe = 5)	= (100/(6 − 1)) * (pain − 2)
Physical function	3	Does your health limit you are walking uphill/climbing stairs	1–3 (yes limited a lot = 1; not limited at all = 3)	= (100/(9 − 3)) * (physical function raw score − 4)
Role function	2	Does your health keep you from getting a job?	1–3 (yes, all the time = 1; none of the time = 3)	= (100/(9 − 3)) * (role function raw score − 2)
Social function	2	Has your physical or emotional health interfered with social activities?	1–5 (not at all = 1; extremely = 5)	= (100/(8 − 2)) * (social function raw score − 2)
Cognitive function	3	Difficulty reasoning or solving problems	1–3 (all of the time = 1; none of the time = 3)	= (100/(9 − 3)) * (cognitive function raw score − 3)
Mental Health	3	In the past 4 weeks, have you felt downhearted and blue?	1–3 (all of the time = 1; none of the time = 3)	= (100/(18 − 3)) * (mental health raw score − 3)
Energy/fatigue	2	In the past 4 weeks, how often have you felt tired?	1–3 (all of the time = 1; none of the time = 3)	= (100/(6 − 2)) * (energy/fatigue raw score − 2)

### Data processing and analysis

All data were imported into STATA version 15® for cleaning and analysis. We conducted descriptive statistics to obtain frequency distributions of socio-demographic and clinical factors aggregated by sex. We tested the hypothesis of differences using Pearson’s Chi-Squared and Fisher’s Exact. The ACTG SF-21 questionnaire administered in the primary study included modifications that deviated from the validated tool as there was a reduction in the number of items scales used to comprehensively assess QOL dimensions. This resulted in the authors conducting an exploratory analysis to examine the structure and the relationship between items, as well as evaluate the construct validity of the item scales. A confirmatory factor analysis on measurement models was conducted by the authors, to confirm whether some of the items that were dropped in the EFA would load in the other factors. Additionally, we were interested in demonstrating fit indices for comparative purposes and to examine modification indices to further elaborate on the model. We acknowledge the use of both EFA and CFA in the same sample is contested and a subject of much ongoing expert debate in an article by Hurley et al. [31] particularly when a small sample size is used.

**Fig. 1:**
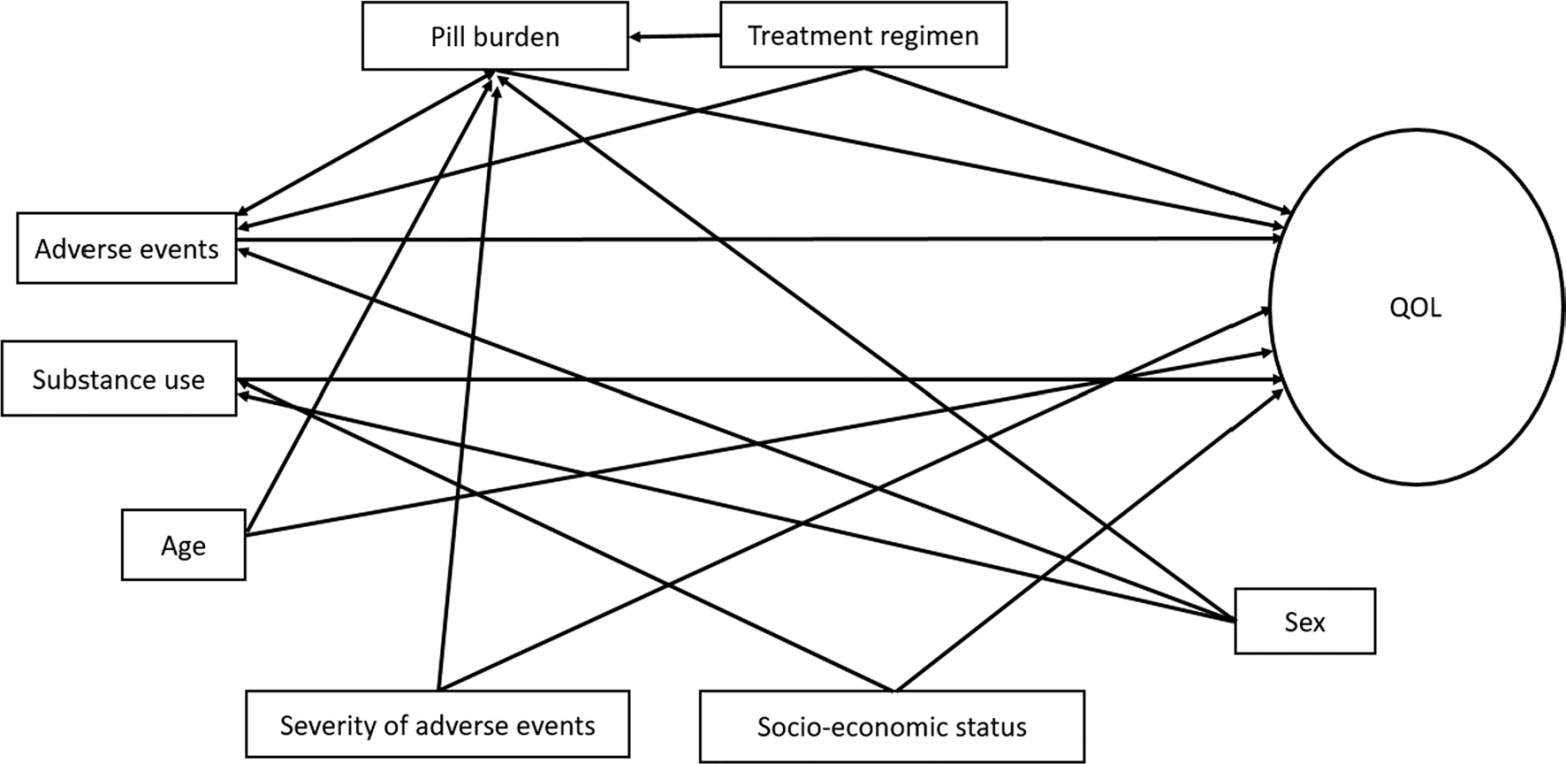
Conceptual model of interrelationships between factors affecting QOL among PLWHA

Structural equation modelling (SEM) analysis guided by the pre-specified a priori framework, was conducted (Fig. 1). It assumed that (a) socio-economic status (SES) directly impacts physical, mental or cognitive QOL and indirectly through substance abuse; (b) adverse effects directly impact physical, mental or cognitive QOL and indirectly through pill burden; (c) treatment regimen directly impacts physical, mental or cognitive QOL and indirectly through pill burden or adverse events (d) sex directly impacts physical, mental and cognitive QOL and indirectly through pill burden or adverse events or substance abuse; (e) age directly impacts physical, mental and cognitive QOL and indirectly through pill burden; (f) severity of adverse events directly impacts physical, mental and cognitive QOL and indirectly through pill burden (g) pill burden directly impacts physical, mental and cognitive QOL and indirectly through adverse events or their severity. The SEM were estimated separately for the three domains. SEM models were estimated and modified by removing all statistically insignificant paths and applying modification indices where they improved model fit as shown in Additional file 1: Appendix B: Table S1 [32, p. 34].

Goodness of fit tests were conducted and different indices were assessed to determine the overall goodness of fit of the final model namely the root mean squared error of approximation (RMSEA) < 0.08, Comparative Fitness Index(CFI) > 0.90, Tucker Lewis Index (TLI) > 0.90, LR chi (likelihood ratio chi-square tests) > 0.05 and root mean squared residual (RMSR) < 0.05 [33, 34].

## Results

### Socio-demographic and behavioural and clinical characteristics of PLH in the study

Sixty-eight percent (199/293) of the sample were women and participants were predominantly of Black African race (99%, 291/293), 92% (270/293) attained secondary education, 75% (220/293) were employed and 56% (164/293) were unmarried. Significantly higher proportions of women attained secondary education, however higher proportions of men were employed and were married, smoked, and consumed alcohol compared to women. There were no significant differences in the proportions of men and women by the different clinical characteristics (Table 2).

**Table 2:** Socio-demographic and behavioural characteristics of PLH in Johannesburg

Demographic characteristics	Total (N = 293)	Male (n = 94)	Female (n = 199)	P value
Age(years)^b^	n (%)	n (%)	n (%)	
18–30	15 (5)	4 (4)	11 (6)	0.003
31–40	129 (44)	29 (31)	100 (50)	
41–50	106 (36)	39 (42)	67 (34)	
51–75	43 (15)	22 (23)	21 (10)	
Mean (sd)	42 (8)	44 (9)	40 (7)	
Education^a,b^				
Primary	23 (8)	12 (13)	11 (6)	0.012
Secondary	246 (84)	77 (82)	169 (85)	
Tertiary	24 (8)	5 (5)	19 (9)	
Employment^b^				
Employed	220 (75)	79 (84)	141 (71)	0.015
Not employed	73 (25)	15 (16)	58 (29)	
Marital status^b^				
Single	164 (56)	33 (35)	131 (66)	< 0.001
Married	129 (44)	61 (65)	68 (34)	
Behavioural characteristic^b^				
Tobacco use				
Non-smoker	277(95)	83 (88)	194 (97)	0.001
Smoker	16 (5)	11 (12)	5 (3)	
Alcohol use^b^				
Yes	84 (29)	34 (36)	50 (25)	0.051
No	209 (71)	60 (64)	149 (75)	
Clinical characteristics				
Treatment regimen^c^				
DRV	144 (49)	49 (52)	95 (48)	0.483
LPV	149 (51)	45 (48)	107 (52)	
Number of adverse events^d^				
0	92 (32)	37 (39)	55 (28)	0.061
1	85 (29)	30 (32)	55 (28)	
2	62 (21)	14 (15)	48 (24)	
3	54 (18)	13 (14)	41 (20)	
Severity of adverse events^d^				
None	92 (31)	37 (39)	55 (28)	0.112
Mild	167 (57)	46 (49)	121 (61)	
Moderate–severe	34 (12)	11 (12)	23 (11)	
Pill burden^d^				
0	41 (14)	15 (16)	26 (13)	0.087
1	29 (10)	14 (15)	15 (8)	
2	43 (15)	15 (16)	28 (14)	
3	37 (13)	11 (12)	26 (13)	
4+	143 (48)	39 (41)	104 (52)	

*N/n* number, *DRV* darunavir, *LPV* lopinavir

^a^Fischer’s exact test

Variables collected at ^b^Screening visit, ^c^Enrolment visit, ^d^over the 48-week period (scheduled and unscheduled visits)

### Exploratory factor analysis (EFA) and confirmatory factor analysis (CFA)

The factor analysis was conducted with the iterated principal axes option and retained three factors [factor (3) option]. Kaiser’s criteria were used to extract factors with eigenvalues greater than 1 as shown in Table 3 and scree plot in Additional file 1: Appendix C: Fig. S2 [35]. In the factor analysis with varimax rotation (minimal loading of 0.3), the 17 items covered 3 discrete dimensions as shown in Additional file 1: Appendix D: Table S2. Eleven items loaded onto Factor 1 and these were items of general health perception, pain, physical function, role function, social function and energy function. Factors 2 and 3 were defined by items measuring the cognitive and mental dimensions respectively. Therefore, the domains identified were physical/functional quality, mental and cognitive. In conducting the confirmatory factor analysis, latent variables were created using factors associated with the specified subset of items during exploratory factor analysis. We used the measurement component of the output to verify that the observed variables load reasonably onto their corresponding latent constructs. In the fourth factor that was dropped, two energy items had loaded and whilst creating the latent variables, collapsed the energy items with physical/functional items and they loaded successfully and items were not lost (Additional file 1: Appendix D). This analysis showed evidence of only one factor between the energy items and the physical/functional items [32, p. 32]. Reasonably, how energetic/fatigued you feel often affects your physical and functional capability. However, collapsing the mental and the cognitive with the physical/functional items was unsuccessful. Latent analysis resulted in three factors confirming the mental, cognitive, and physical/functional domains therefore they were analysed as three separate latent variables.

**Table 3:** Exploratory factor analysis

Factor	Eigenvalues
Factor 1	6.208
Factor 2	2.271
Factor 3	1.310
Factor 4 (dropped)	0.7475

### SEM and pathway analysis of physical and functional QOL

**Fig. 2:**
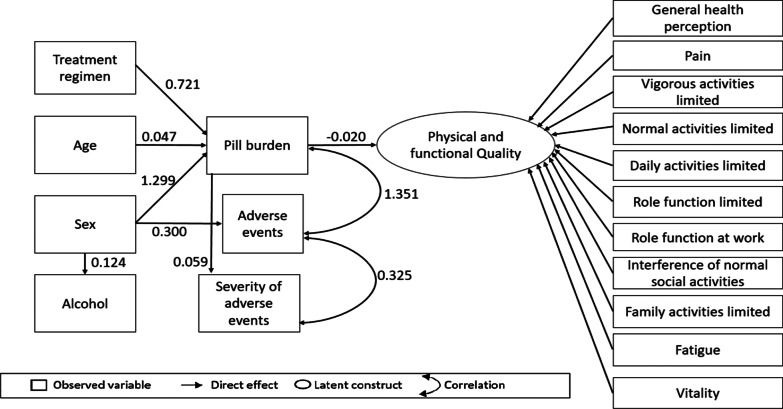
Final model of factors influencing physical and functional QOL among PLH receiving second-line ART in Johannesburg

Figure 2 shows the final SEM path model that depicts the inter-relationships between variables and physical and functional QOL. Table 4 shows the statistical output of the SEM model. Age, sex, and treatment regimen had in-direct effects on physical and functional QOL which were mediated by pill burden. The model had a moderate fit, as the likelihood ratio test did not meet the criteria for a well-fitted model. However, we proceeded based on the chi squared statistic having no longer completely relied upon as a bases of rejection or acceptance as it is shown to very sensitive to sample size and normality of data [33, 36, 37]. (CFI 0.982; TLI 0.980 RMSEA 0.038; RMSR 0.052 and LR chi2 0.002) (Table 4, Fig. 2).

**Table 4:** Statistically significant latent structure, direct and indirect effects on physical QOL

Latent variables	Estimate	STD	SE	P-value
General health perception → physical/functional QOL	1.220	0.452	0.148	< 0.001
Pain → physical/functional QOL	1.613	0.438	0.206	< 0.001
Vigorous activities limited → physical/functional QOL	1	0.931		
Normal activities limited → physical/functional QOL	0.953	0.925	0.034	< 0.001
Daily activities limited → physical/functional QOL	0.659	0.836	0.032	< 0.001
Role function limited → physical/functional QOL	0.959	0.974	0.028	< 0.001
Role function at work → physical/functional QOL	1.008	0.999	0.025	< 0.001
Interference of normal social activities → physical/functional QOL	0.968	0.423	0.120	< 0.001
Family activities limited → physical/functional QOL	0.525	0.413	0.069	< 0.001
Fatigue → physical/functional QOL	0.515	0.295	0.114	< 0.001
Vitality → physical/functional QOL	0.551	0.262	0.105	< 0.001
Direct effects				
Sex → alcohol use	0.124	0.118	0.061	0.044
Sex → adverse events	0.300	0.130	0.1.22	0.014
Pill burden → physical/functional QOL	− 0.020	− 0.219	0.006	0.001
Age → pill burden	0.047	0.156	0.020	0.020
Treatment → pill burden	0.722	0.122	0.311	0.029
Sex → pill burden	1.298	0.185	0.401	0.001
Pill burden → severity of adverse events	0.059	0.356	0.011	< 0.001
Indirect effects				
Age → pill burden → severity of adverse events	0.003	0.056	0.001	0.030
Treatment regimen → pill burden → severity of adverse events	0.043	0.043	0.020	0.032
Sex → pill burden → severity of adverse events	0.077	0.066	0.028	0.006
Age → pill burden → physical/functional QOL	− 0.0009	− 0.063	0.0004	0.054
Treatment regimen → pill burden → physical/functional QOL	− 0.014	− 0.097	0.007	0.055
Sex → pill burden → physical/functional QOL	− 0.025	− 0.034	0.011	0.019
Correlations				
Number of adverse events ↔ severity of adverse events	0.325	0.537	0.040	< 0.001
Number of adverse events ↔ pill burden	1.351	0.513	0.210	< 0.001

### SEM and pathway analysis for cognitive QOL

**Fig. 3:**
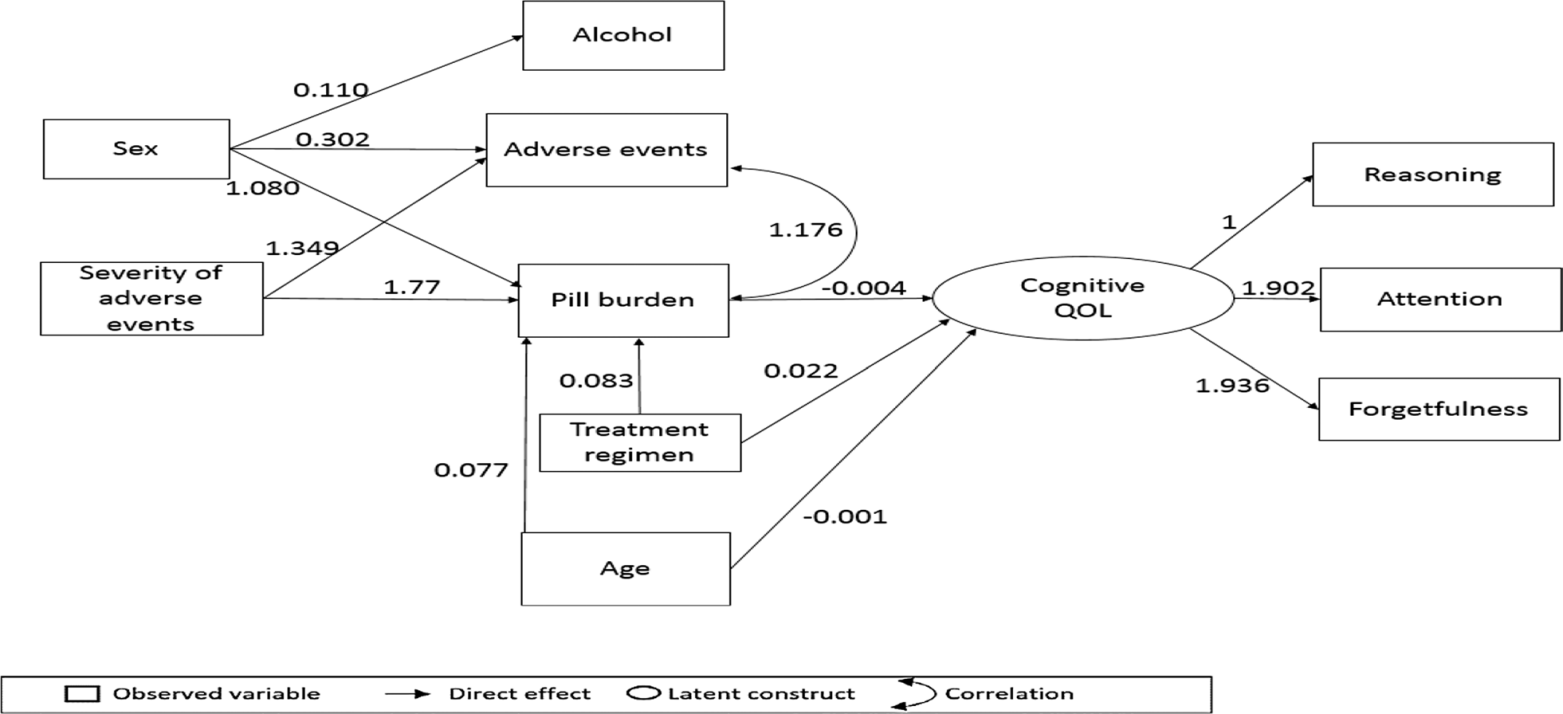
Final model of factors influencing cognitive QOL among PLH receiving second-line ART in Johannesburg

Figure 3 shows the final SEM path model that depicts the inter-relationships between variables and cognitive QOL. Table 5 shows the statistical output of the model. Age and treatment regimen had direct effects on cognitive QOL, and indirect effects mediated by pill burden. Pill burden also mediated the relationships of sex and severity of adverse effects on cognitive QOL. The model had a good fit as all the criteria for a well-fitted model were met (CFI 0.992; TLI 0.987; RMSEA 0.025; RMSR 0.034 and LR chi2 0.235).

**Table 5:** Statistically significant latent structure, direct and indirect effects on cognitive QOL

Latent variables	Estimate	STD	SE	P-value
Reasoning → cognitive QOL	1	0.718		
Attention → cognitive QOL	1.902	0.900	0.180	0.000
Forgetfulness → cognitive QOL	1.937	0.635	0.196	0.000
Direct effects				
Pill burden → cognitive QOL	− 0.004	− 0.177	0.001	0.005
Age → cognitive QOL	− 0.001	− 0.133	0.006	0.046
Treatment regimen → cognitive QOL	0.023	0.157	0.009	0.013
Age → pill burden	0.077	0.192	0.019	0.000
Treatment regimen → pill burden	0.830	0.130	0.320	0.008
Severity of adverse events → pill burden	1.775	0.349	0.266	0.000
Sex → pill burden	1.080	0.158	0.363	0.003
Sex → adverse events	0.302	0.107	0.124	0.015
Severity of adverse events → adverse events	1.349	0.642	0.093	0.000
Sex → alcohol	0.110	0.114	0.056	0.049
Indirect effects				
Age → pill burden → cognitive QOL	− 0.0003	− 0.015	0.0001	0.023
Treatment regimen → pill burden → cognitive QOL	− 0.003	− 0.047	0.002	0.050
Sex → Pill burden → cognitive QOL	− 0.004	− 0.019	0.002	0.042
Severity of adverse events → pill burden → cognitive QOL	− 0.007	− 0.005	0.003	0.010
Correlations				
Number of adverse events ↔ pill burden	1.176	0.419	0.258	0.0000

### SEM and pathway analysis of mental QOL

**Fig. 4:**
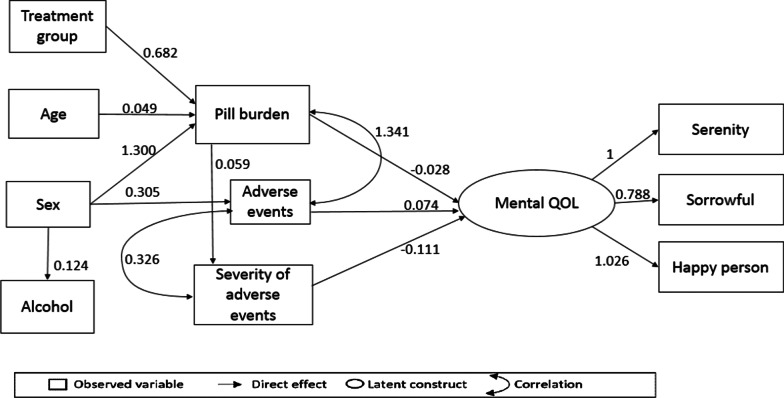
Final model of factors influencing mental QOL among PLH receiving second-line ART in Johannesburg

Figure 4 shows the final SEM path model that depicts the inter-relationships between variables and mental QOL. Table 6 shows the statistical output of the model. Severity of adverse events had a direct effect on Mental QOL. Sex had an indirect effect on mental QOL and was mediated by adverse events and pill burden. Age and treatment regimen had indirect effects on Mental QOL and were mediated by pill burden. The model had a good fit as all the criteria for a well-fitted model were met (CFI 1.000; TLI 1.008; RMSEA 0.000; RMSR 0.034 and LR chi2 0.673).

**Table 6:** Statistically significant latent structure, direct and indirect effects on Mental QOL

Latent variables	Estimate	STD	SE	P-value
Serenity → mental QOL	1	0.912		
Despondent → mental QOL	0.788	0.815	0.042	< 0.001
Generally happy → mental QOL	1.027	0.977	0.042	< 0.001
Direct effects				
Sex → alcohol use	0.124	0.118	0.061	0.044
Sex → adverse events	0.262	0.132	0.203	0.022
Pill burden → mental QOL	− 0.028	− 0.221	0.010	0.010
Severity of adverse events → mental QOL	− 0.111	− 0.155	0.056	0.019
Adverse events → mental QOL	0.074	0.189	0.035	0.020
Age → pill burden	0.049	0.161	0.020	0.014
Treatment → pill burden	0.682	0.118	0.313	0.029
Sex → pill burden	1.300	0.184	0.402	0.001
Pill burden → severity of adverse events	0.059	0.356	0.011	< 0.000
Indirect effects				
Age → pill burden → mental QOL	− 0.001	− 0.060	0.0009	0.072
Treatment regimen → pill burden → mental QOL	− 0.021	− 0.103	0.012	0.092
Sex → pill burden → mental QOL	− 0.287	− 0.037	0.141	0.041
Sex → adverse events → mental	0.014	0.025	0.018	0.435
Pill burden → severity of adverse events → mental	− 0.014	− 0.201	0.010	0.215
Correlations				
Number of adverse events ↔ severity of adverse events	0.326	0.539	0.040	< 0.001
Number of adverse events ↔ pill burden	1.341	0.510	0.209	< 0.001

## Discussion

Using 48-week data from an RCT, this study aimed to determine the inter-relationships between clinical factors, behavioural, socio-demographic variables and QOL among PLH receiving second-line ART. Confirmatory factor analysis yielded only three distinct domains: (a) namely physical and functional, (b) mental and, (c) cognitive, as opposed to the standard eight QOL domains. Socio-demographic factors of age and sex indirectly impacted on QOL. Treatment regimen, pill burden and severity of adverse events (all clinical factors) were important mediators, each of which differentially impacted on the different QOL domains.

PLH on lopinavir reported higher cognitive QOL compared to those on darunavir but there were no significant differences between the treatment groups on physical function and mental QOL. There is strong supportive evidence that darunavir has sustained high rates of viral suppression alongside a better side effect profile i.e., better metabolic profile, and lower impact on lipids [38]. In contrast, there have been studies that show that patients on lopinavir have commonly reported metabolic concerns [39–41]. Gupta et al., in a study observing the effect of lopinavir on mice, reported that these metabolic abnormalities largely contributed to decreases in cognitive function [40]. However, the relevance of this study to humans is currently unknown.

Treatment regimen was associated with pill burden but did not have effects on adverse effects-although pill burden covaried with adverse effects. The co-relations of pill burden and adverse events are consistent with existing literature [14]. Previous research found that treatment of adverse events secondary to ART increases pill burden [14, 42, 43]. Furthermore, since darunavir has lower dosage compared to lopinavir it is more tolerable and safer [39, 44]. This suggests a lower incidence of adverse events and consequently, less medication required to treat them. The CASTLE study in the United States reported that patients on darunavir had less gastrointestinal adverse events compared to patients on lopinavir [45]. However, in our study, we did not find an association between treatment regimen and adverse effects and could be a result of smaller sample size as compared to the CASTLE study.

Pill burden mediated the effects of age, sex, and treatment regimen on all three QOL domains. Increase in age was associated with increased pill burden and decreased QOL. The direct effects of age on pill burden and ultimately reduced QOL can be explained by the expected higher prevalence of co-morbidities among older PLH which in turn increases the number of medications, or pill burden, ultimately impacting on QOL [46]. In other studies, long-term exposure to treatment has been linked with increasing pill burden among older patients [42].

Sex impacted on QOL, but these relationships were influenced by clinical and behavioural factors. Women reported a higher number and severity of adverse events, higher pill burden and lower scores in all three QOL domains compared to men. This is consistent with Pereira et al.’s study which found that the incidence of HIV-related co-morbidities and associated pill burden was higher amongst women [3]. Moreover, the high number of adverse events reported amongst women had an unexpected positive association with Mental QOL scores. The findings of this current study do not support previous research [3, 47]. Wouters et al. conducted a structural equation model in South Africans on long-term changes in the physical and emotional QOL of patients on ART and reported that adverse events were negatively associated with emotional QOL [47]. It is difficult to explain this relationship between adverse events and mental QOL, however, it is possible that the response and care that a study participant receives in the event of an adverse effect, may paradoxically contribute positively to their mental QOL. This would mean that, in a study setting, a patient may feel more valued and supported, and as a consequence this would impact positively on their mental well-being.

Sex also had indirect impacts on QOL that were mediated by alcohol or substance use. Male PLH were more likely to engage in alcohol or substance use which lowered QOL. Previous studies have shown that alcohol (ab)use is associated with aggravating co-morbidities, delayed initiation to ART, adherence difficulties and decreased QOL [48, 49].

This study shows that a reduction in QOL may result from a myriad of factors including age, sex, HIV treatment, and pill burden which covary with adverse events and behavioural factors. These factors must be considered when reviewing treatment options and developing medication regimens. The public health implications of this study’s findings should be contemplated because as PLH age, the impact of co-morbidities and pill burden as it related to QOL becomes compelling.

This study has several limitations. Firstly, the confirmatory analysis confirmed that data only loaded onto three QOL domains instead of the eight expected domains. It is possible that this could be the result of the primary study’s used of a shorter version/fewer items of the ACTG SF-21 questionnaire instead of the full scale. The loading of data onto only three QOL domains limits the study’s comparability to studies that used the full scale and eight QOL domains. Considering that the primary study was an open label RCT. There was potential for measurement biases including response bias i.e., a tendency for patients to report symptoms in a way they think is expected; as well as interviewer bias where a research assistants’/nurses’ knowledge may influence the structure and manner with which questions are administered and may subtly influence responses.

The sample is a volunteer sample within a clinical trial setting therefore the external validity of the results is limited. The number of ART pills were not included as part of the pill burden but was adequately adjusted for in the SEM analysis through the inclusion of regimen as a variable. Lastly, as a secondary study there are limitations due to the reliance on only those variables collected as part of the primary study. Variables which may be important in understanding QOL for example psycho-social factors, family support, level of health education, stigma, and history of ART including time on first-line treatment, adherence data and frequency/dosage of alcohol and tobacco use were not measured therefore are not considered in analyses.

## Conclusions

Our study showed that socio-demographic and clinical factors affect QOL among PLH in a South African trial setting. QOL was lower amongst participants with a higher pill burden, females and older age group. This study has implications for HIV management. It points to a need for considering the risk/benefit ratio of medications before initiating them. Furthermore, this study revealed the impact of co-morbidities and pill burden amongst the older participants with HIV, revealing the importance of guided functional and public health services that need to be tailored for the needs of the aging HIV population. The study supports the evidence that darunavir has a better side effects profile and should be considered when switching to second line therapy to improve QOL.

Females report a higher number of pill burden and adverse events as compared to males, as a result, are vulnerable in experiencing lower mental, physical and cognitive QOL. Therefore, to improve QOL of PLH, gender differences should be acknowledged.

## Supplementary Information

**Additional file 1: Appendix A.** QOL questionnaire. **Appendix B: Table S1.** Model fit before and after Modification Indices (MI). **Appendix C: Figure S1.** Eigenvalues Scree Plot. **Appendix D: Table S2.** Exploratory standardized factor loadings.

## Data Availability

The data that support the findings of this study are available from Ezintsha but restrictions apply to the availability of these data, which were used under license for the current study, and so are not publicly available. Data are however available from the authors upon reasonable request and with permission of Ezintsha.
