# Supplementary material for: Quality of life and associated factors among people receiving second-line anti-retroviral therapy in Johannesburg, South Africa
Source: BMC Infect Dis. 2022 May 12;22:456. doi: 10.1186/s12879-022-07429-9 (PMC9103409; doi:10.1186/s12879-022-07429-9)
Supplement: Supplementary file 1 — Additional file 1: Appendix A. QOL questionnaire. Appendix B: Table S1. Model fit before and after Modification Indices (MI). Appendix C: Figure S1. Eigenvalues Scree Plot. Appendix D: Table S2. Exploratory standardized factor loadings. [file 12879_2022_7429_MOESM1_ESM.docx]

# APPENDICES

**Appendix A: QOL questionnaire**


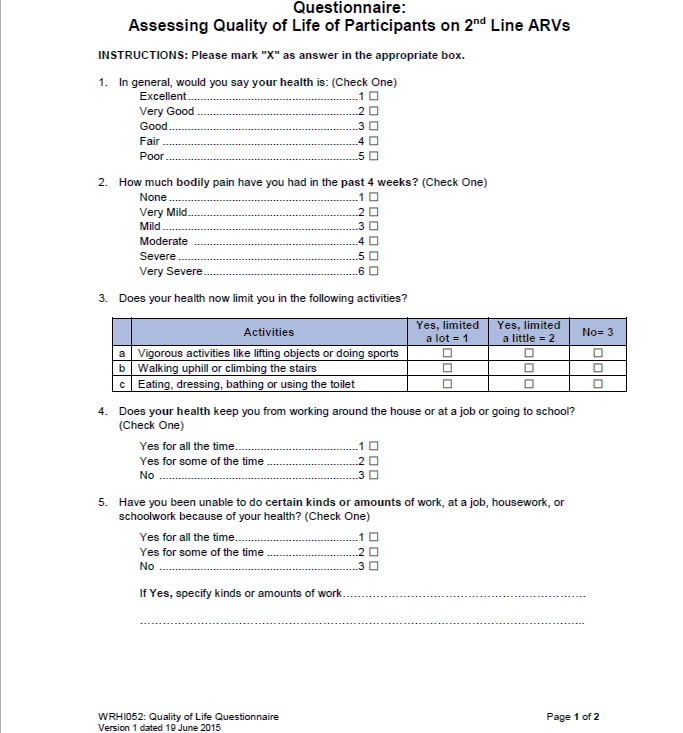


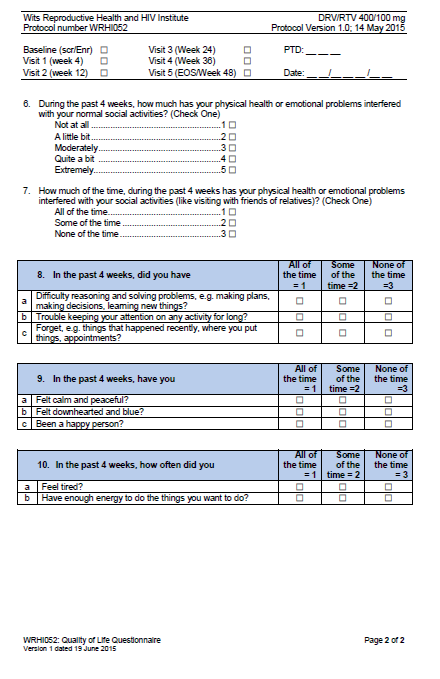


**Appendix B: Table S1. Model fit before and after Modification Indices (MI)**

| **Physical funtional QOL** | | | | |
| --- | --- | --- | --- | --- |
|  | Criteria for good fit | Before MI |  | After MI |
| CFI | >0.90 | 0.909 | The following pathways were removed: adverse events and physical/functional QOL; pill burden and adverse events; age and physical/functional QOL; sex and physical/functional QOL as well as treatment regimen and adverse events.  Following pathways were added: correlation between pill burden and adverse events; correlation between adverse events and severity of adverse events. | 0.982 |
| TLI | >0.90 | 0.891 |  | 0.980 |
| RMSEA | <0.08 | 0.089 |  | 0.038 |
| RMSR | <0.05 | 0.086 |  | 0.052 |
| LR chi2 | >0.05 | 0.000 |  | 0.001 |
| **Cognitive QOL** | | | | |
|  | Criteria for good fit | Before MI | The following pathways were removed: adverse events and cognitive QOL; treatment regimen and adverse events; pill burden and adverse events; sex and cognitive QOL.  Following pathways were added: correlation between Pill burden and adverse events | After MI |
| CFI | >0.90 | 0.893 |  | 0.992 |
| TLI | >0.90 | 0.819 |  | 0.987 |
| RMSEA | <0.08 | 0.096 |  | 0.025 |
| RMSR | <0.05 | 0.050 |  | 0.034 |
| LR chi2 | >0.05 | 0.000 |  | 0.235 |
| **Mental QOL** | | | | |
|  | Criteria for good fit | Before MI | The following pathways were removed: treatment regimen and adverse events; sex and mental QOL; age and mental QOL were removed.  Following pathways were added:  correlation between Pill burden and adverse events and correlation between adverse events and severity of adverse events. | After MI |
| CFI | >0.90 | 0.784 |  | 1.000 |
| TLI | >0.90 | 0.665 |  | 1.008 |
| RMSEA | <0.08 | 0.171 |  | 0.000 |
| RMSR | <0.05 | 0.132 |  | 0.028 |
| LR chi2 | >0.05 | 0.000 |  | 0.780 |

**Appendix C: Scree plot**

Figure S1: Eigenvalues Scree Plot

**Appendix D: Table S2. Exploratory standardized factor loadings**

| **Domains** | **Items** | **Factor 1** | **Factor 2** | **Factor 3** |
| --- | --- | --- | --- | --- |
| **General health** | Item 1 | 0.4420 |  |  |
| **Pain** | Item 2 | 0.3857 |  |  |
| **Physical function** | Item 3 | 0.8983 |  |  |
|  | Item 4 | 0.8516 |  |  |
|  | Item 5 | 0.8597 |  |  |
| **Role function** | Item 6 | 0.9972 |  |  |
|  | Item 7 | 0.9970 |  |  |
| **Social function** | Item 8 | 0.4568 |  |  |
|  | Item 9 | 0.5030 |  |  |
| **Energy** | Item 10 | 0.7904 |  |  |
|  | Item 11 | 0.4626 |  |  |
| **Cognitive function** | Item 12 |  |  | 0.7237 |
|  | Item 13 |  |  | 0.9064 |
|  | Item 14 |  |  | 0.6334 |
| **Mental Health** | Item 15 |  | 0.9122 |  |
|  | Item 16 |  | 0.8081 |  |
|  | Item 17 |  | 0.9669 |  |
